# Treatment of meningioma and glioma with protons and carbon ions

**DOI:** 10.1186/s13014-017-0924-7

**Published:** 2017-12-01

**Authors:** Sebastian Adeberg, Semi B. Harrabi, Vivek Verma, Denise Bernhardt, Nicole Grau, Jürgen Debus, Stefan Rieken

**Affiliations:** 1 Department of Radiation Oncology, University Hospital of Heidelberg, Im Neuenheimer Feld 400, 69120 Heidelberg, Germany; 2 Clinical Cooperation Unit Radiation Oncology, German Cancer Research Center (DKFZ), Im Neuenheimer Feld 280, 69120 Heidelberg, Germany; 3 Heidelberg Ion-Beam Therapy Center, Im Neuenheimer Feld 450, 69120 Heidelberg, Germany; 4 Heidelberg Institute for Radiation Oncology (HIRO), National Center for Radiation Research in Oncology (NCRO), 69120 Heidelberg, Germany; 5 Department of Radiation Oncology, University of Nebraska Medical Center, 505 S 45th Street, Omaha, NE 68106 USA

**Keywords:** Ion beam radiation therapy, Glioma, Proton beam therapy, Radiotherapy, Meningioma

## Abstract

The rapid rise of particle therapy across the world necessitates evidence to justify its ever-increasing utilization. This narrative review summarizes the current status of these technologies on treatment of both meningiomas and gliomas, the most common benign and malignant primary brain tumors, respectively. Proton beam therapy (PBT) for meningiomas displays high rates of long-term local control, low rates of symptomatic deterioration, along with the potential for safe dose-escalation in select (but not necessarily routine) cases. PBT is also associated with low adverse events and maintenance of functional outcomes, which have implications for quality of life and cost-effectiveness measures going forward. Data on carbon ion radiation therapy (CIRT) are limited; existing series describe virtually no high-grade toxicities and high local control. Regarding the few available data on low-grade gliomas, PBT provides opportunities to dose-escalate while affording no increase of severe toxicities, along with maintaining appropriate quality of life. Although dose-escalation for low-grade disease has been less frequently performed than for glioblastoma, PBT and CIRT continue to be utilized for the latter, and also have potential for safer re-irradiation of high-grade gliomas. For both neoplasms, the impact of superior dosimetric profiles with endpoints such as neurocognitive decline and neurologic funcionality, are also discussed to the extent of requiring more data to support the utility of particle therapy. Caveats to these data are also described, such as the largely retrospective nature of the available studies, patient selection, and heterogeneity in patient population as well as treatment (including mixed photon/particle treatment). Nevertheless, multiple prospective trials (which may partially attenuate those concerns) are also discussed. In light of the low quantity and quality of available data, major questions remain regarding economic concerns as well.

## Background

Gliomas are the most frequent primary brain malignancies, and are a diverse constellation of disease ranging from relatively indolent (World Health Organization (WHO) Grade 1 pilocytic astrocytoma) to the almost universally fatal glioblastoma (WHO grade IV). These also encompass the equally diverse low-grade gliomas (LGGs, WHO grade II) and anaplastic gliomas (WHO grade III). Although the prognosis of gliomas varies based on grade and molecular signature [1], among other factors, a common element of delivering radiation therapy (RT) to these neoplasms is the necessity to spare surrounding organs-at-risk (OARs) from RT dose. To this extent, the emerging modality of particle therapy, chiefly comprised of proton beam therapy (PBT) and carbon ion RT (CIRT), are appealing. The signature Bragg peak of both beams result in reduced dose distal to the target of interest, together with a relatively narrow lateral penumbra, thus sparing adjacent OARs to a greater degree as compared with photon RT [2].

Meningiomas account for 15–20% of all primary brain tumors in adults, and are the most common benign primary neoplasm of the brain [3]. Tumor classification is highly meaningful, as early RT may be withheld in low-grade disease, whereas higher-grade meningiomas (atypical and malignant disease) may benefit from immediate RT [4]. The high survival associated with meningiomas leads to an increased emphasis on sparing adjacent OARs, in efforts to maintain neuronal functionality and quality of life (QOL) in a population that may experience substantial detriment if this is not achieved.

The use of PBT and CIRT is rapidly rising across the world, and the implementation of this technology has outpaced the completion of prospective trials that support its utility. The goal of this review is to highlight the existing data of PBT and CIRT in meningioma and adult glioma, as well as postulate future applications and implications for prospective studies going forward.

## Meningioma

PBT for meningioma displays superior dosimetric profiles as compared to photon-based RT. A planning study of 10 patients utilizing conventionally-fractionated RT showed decreased doses to bilateral hippocampi, cochleae, and whole brain, among many other structures [5]. Although most cases in the photon group were intensity-modulated RT (IMRT), there were a couple patients receiving 3D conformal RT (3DCRT). Nevertheless, the study also estimated that the risk of secondary radiation-induced malignancies could be halved with the use of PBT.

Clinically, PBT has been utilized to treat meningiomas since the early 1980s, albeit with non-modern technology, imaging, and planning tools. Nevertheless, these reports have accrued long-term follow-up, demonstrating expectedly high 5-year recurrence-free and overall survival (OS) figures of 100% and 93%, respectively [6]. Photon data illustrate local control (LC) rates of 91% at 10 years for benign meningiomas and 81% at 5 years and 53% at 10 years for high-grade disease [3]. A more contemporary report of PBT for meningioma is largely associated with critical anatomical areas such as the skull base [7]. These utilized either single-fraction PBT radiosurgery (*n* = 18) or hypofractionated (3-fraction, *n* = 5) PBT, and demonstrated 100% LC at median 31 months follow-up in patients treated with PBT radiosurgery. The LC was 88% in the five patients undergoing hypofractionated therapy, likely a consequence of the larger volume of disease treated with fractionation.

Long-term data presented by the Centre de Protonthérapie d’Orsay support these results [8]. The investigators utilized combined photon (2/3 of the total dose) and proton (1/3 of the total dose) therapy, while displaying the ability of PBT-mediated dose escalation with a median dose of 61 Gy relative biological effectiveness (RBE) and observed a 4-year LC rate of 88%. This is encouraging in light of the inclusion of atypical and anaplastic histologies. Importantly, the group published a secondary analysis displaying that PBT affords low adverse events and maintenance of functional outcomes following PBT, which has high implications for QOL [9]. This group’s work was updated (*n* = 24) with more novel techniques as well as utilization of a more balanced ratio of photons to protons (mean doses 30.96 and 34.05 Gy(RBE), respectively), displaying several findings. The most important was the association of total dose with survival, adding further significance to the notion of dose-escalation [10]. This finding is in line with a recent publication from Indiana University. Despite including patients treated in the adjuvant and nonoperative setting, the authors determined that doses of over 60 Gy(RBE) were associated with a 5-year LC of 88%, as compared to just 50% with doses ≤60 Gy(RBE) (*p* = 0.038) [11]. However, that association was not evaluated on multivariable analysis; it thus could have been likely that larger tumors (which are more likely to recur) received lower doses on account of their size. Nevertheless, the concept of safe dose-escalation must be further explored; if proven, it would give particle therapy a major advantage insofar as permitting safer dose–escalation [12].

Other institutions’ publications have also added to the encouraging safety and efficacy profiles of PBT for meningiomas. The Harvard experience from 1996 to 2007 (*n* = 50) evaluated a single fraction of 13 Gy (RBE), with just under two-thirds of cases being primary/nonoperative [13]. The 3-year LC was estimated at 94%, with low rates of RT-associated morbidity; symptomatic worsening occurred in less than 10% of patients. Next, an updated report from the Paul Scherrer Institute of 32 patients, mostly treated in the postoperative setting, and a median dose of 56 Gy(RBE), described long-term outcomes with a mean follow-up of 62 months [14]. The treatment was tolerated well, with 5-year LC of 85%, partially attributed to the higher proportion of postoperative cases and grade I disease, among other salient factors. Lastly, a large (*n* = 72) experience of cavernous sinus meningiomas from Loma Linda University demonstrated excellent 5-year LC rates of 96% for benign histology and 50% for atypical histology [15]. Although most patients were grade I, a token observation was that larger volumes of disease were still satisfactorily controlled. Therein, 5-year LC was 100% in patients with disease ≤20 cm^3^ versus 95% with tumors over 20 cm^3^. Importantly, RT-induced optic toxicities were restricted to just three patients, all of whom had direct optic nerve involvement and hence received full dose.

Studies of carbon ion irradiation for meningiomas are limited to single-institution retrospective reports lumping these cases with other histologies and/or co-administration of photon-based RT [16, 17]. However, existing data of atypical/anaplastic meningiomas after mixed photon-carbon ion treatment (median 50.4 Gy and 18 Gy(RBE), respectively) with long-term follow-up (median 77 months) show high rates of primary local control (86% at 5 years) with virtually no high-grade adverse events [12]. Irradiation with photons followed by a carbon ion boost in the recurrent setting also displays a 67% rate of local control at 1 year, with no high-grade toxicities [18]. This question is currently addressed in the MARCIE trial at the Heidelberg Ion-Beam Therapy Center (HIT) where subtotally resected high-grade meningioma receive a carbon ion boost with 16 Gy(RBE) in 3 Gy(RBE) fractions combined with an IMRT base plan of 48-52Gy [19].

There are several analyses and reflections in light of the overall few existing data for meningioma. Although PBT has displayed encouraging tumor control rates with low risk of adverse events, it is important to contextualize these data. There are multiple discrepancies between the studies discussed herein, including proportion of patients undergoing surgery prior to RT, numbers with atypical or malignant disease, and the large range of irradiated tumor volumes. However, because RT for meningioma is commonly restricted to patients who are unsuitable for surgery or incomplete surgical resection, it is somewhat necessary to lump heterogeneous patients into a series. It is, nevertheless, of paramount importance to critically recognize whether particle therapy actually improves on outcomes of photon RT for high-risk meningiomas. To this extent, an additional limitation of these retrospective studies is clearly related to patient eligibility and selection, which limits applicability to other cohorts and overall generalization. Additionally, although it is agreed that particle therapy offers safer ability to dose-escalate the tumor while maintaining low doses to OARs, the role of dose-escalation must be better defined going forward. This is especially true given the utility of, and recent increase in, intensity-modulated proton therapy (IMPT), which can be even more conformal than forward-planned PBT [20, 21] or single beam optimized proton plans. Lastly, it remains unresolved whether OAR sparing from the biophysical advantages of PBT translate into improved clinical outcomes. It is also imperative to provide long-term functional and QOL data for this neoplasm, which may directly impact the perception of particle therapy by patients and payers.

## Glioma

Because PBT affords lower integral brain doses, its dosimetric improvements as compared to IMRT may be notable in an otherwise largely healthy population of patients with low-grade gliomas that are expected to achieve long-term cure with RT-based therapy [22]. For both LGGs and high-grade gliomas (HGGs), dosimetric results have predictably shown a dose reduction to nearby OARs, particularly those farther away from the target [23–25]. These areas include the hippocampi, subventricular zones, hearing and visual apparatus, and pituitary gland. It has also been postulated, similar to the aforementioned analogous data in meningiomas, that PBT roughly halves the risk of developing RT-induced neoplasms as compared with photon-based therapies, owing to the decreased dose to the whole brain [24], even though this is of comparatively less importance in HGGs. Late effects were also studied by Karunamuni et al., who found a temporal lobe-pronounced dose-dependent cortical thinning of 0.0033 mm per Gy [26], which could relate to the higher likelihood of dementia observed after long-term follow up after radiotherapy [27, 28]. Hence, dose reductions to potentially every one of the aforementioned areas have important implications for the maintenance of QOL and cost-effectiveness following curative-intent RT, but data are lacking to support this notion.

Owing to the relative rarity of LGG, the overall volume of data is comparatively less extensive. However, a distinct advantage of the available data is the prospective nature of multiple investigations (discussed subsequently).The largest study to date, an unpublished retrospective analysis of 58 patients from the Proton Collaborative Group registry, illustrated no grade ≥ 3 toxicities when treated with up to 54 Gy(RBE) (this work did not ascertain clinical outcomes) [29]. The initial Harvard phase I/II experience (*n* = 20: *n* = 7 LGG, *n* = 13 HGG) demonstrated several notable findings [30]. First, the ability to dose-escalate was again apparent, as exemplified by the cumulative prescribed doses to LGGs and anaplastic gliomas of 68.2 and 79.7 Gy(RBE), respectively. With five-year follow-up, despite the fact that just nine patients received PCV chemotherapy, 5-year OS was a remarkable 71% (although it is acknowledged that salvage treatments may impact this figure). Despite the similarities with contemporary data, treatment incurred more adverse events than those afforded by lower doses [22].

A prospective QOL study of 20 patients with LGG was notable for assessing a diverse array of QOL measures at many subsequent time points [31]. With a median follow-up of 5.1 years, there were no declines in several neurocognitive QOL parameters, along with statistical improvements in QOL scores for fatigue and visuospatial parameters. This study had notable limitations, including a relatively heterogeneous cohort comprised of both primary (*n* = 8) and recurrent (*n* = 12) LGGs, as well as patients with prior symptomatology leading to PBT initiation (thus, a potentially altered baseline). Patients that progressed were also removed from the study, and QOL for those patients was not included. The group expanded upon these results by illustrating the impact of tumor location on improvement in neuropsychological testing at long-term follow-up [32].

Initial evaluation of PBT for glioblastoma was chiefly in the context of safe dose-escalation. In a phase II study of 23 patients receiving 90 Gy(RBE) (57.6 Gy(RBE) of which was delivered with PBT), the median OS was highly encouraging at 20 months [30]. However, patterns of failure analysis demonstrated that most recurrences remained in-field. Thirty percent of patients experienced radiation necrosis with such high doses.

Dose-escalation for glioblastoma, by means of a hyperfractionated concomitant boost technique, was echoed by both retrospective and prospective reports from the University of Tsukuba [33, 34]. Concomitantly with delivery of 50.4 Gy in 28 fractions with photons, 23.4 Gy(RBE) to a coned-down volume was administered for the first half of treatment; in the second half, the same boost dose was given to the entire initial volume. Thus, the cumulative dose was 96.6 Gy(RBE) in 56 total fractions. Of 20 patients, there were two cases of nonhematologic grades ≥3 toxicity (leukoencephalopathy and radiation necrosis), and median OS was 22 months.

Even though the first dose-escalation studies with particles showed promising results [34–36], there are currently no high-level data that substantiate the benefit of dose escalation in this setting [37]. This question will in part be addressed by the prospective CLEOPATRA trial at the Heidelberg Ion-Beam Therapy Center (HIT). After receiving a photon base plan of 50.0 Gy, patients are randomized to a proton boost (up to 10.0 Gy(RBE) in 5 fractions) versus carbon ions (escalating doses up to 18.0 Gy(RBE) in 6 fractions) [38]. Retrospective data of this approach using 50.0Gy base plans followed by a 10Gy(RBE) proton boost plan on a reduced target volume revealed at least equivalent acute and chronic toxicity rates compared to standard photon plans (60.0Gy in 2Gy fractions), achieving similar progression and survival rates [39]. These results are appealing since smaller target volumes might be associated with improved QOL, neurocognitive- and neuronal function.

Next, because both LGGs and HGGs may recur, a retrospective investigation evaluated re-irradiation of 26 diverse cerebral cases, 8 of which were re-treated with PBT (*n* = 5 glioblastoma, *n* = 1 anaplastic glioma, n = 1 ependymoma, n = 1 meningioma) [40]. The median dose of initial photon RT was 55 Gy, and the median interval to re-treatment was 16 months in all patients. The median re-irradiation dose was relatively low (33 Gy(RBE)), which is important to understand in the context of no observed grade ≥ 2 toxicities and two cases of uncomplicated radiation necrosis. Median OS in the PBT re-treated patients was 19.4 months, which the authors reported as favorable compared to existing photon literature.

Two phase I/II trials from Chiba University will be described pertaining to CIRT. First, an investigation of 48 (*n* = 16 anaplastic, *n* = 32 glioblastoma) gliomas consisted of treatment with 50 Gy of conventionally-fractionated photon RT with an 8-fraction CIRT boost (dose ranging from 16.8 to 24.8 Gy(RBE)) with concurrent nimustine chemotherapy. The authors observed no grade ≥ 3 toxicities, with median OS of 35 months in grade III disease and 17 months in glioblastoma. Notably, the median progression-free survival (PFS) and OS in patients treated with the highest boost doses was 14 and 26 months, respectively [41]. Next, the same working group described a more uniform population of 14 diffuse grade II astrocytoma cases treated with CIRT (46.2–50.4 Gy(RBE) or 55.2 Gy(RBE)) [36]. Concomitant chemotherapy was not routinely utilized, but was performed for select salvage cases. Of the five patients treated to 55.2 Gy(RBE), median PFS and OS were 91 months and not reached, respectively; corresponding figures for the remaining 9 patients were 18 and 28 months. Although these numbers are clearly encouraging, the causes of the major differences in survival between the lower-dose and dose-escalated cohorts is unclear. Although four patients developed grade 3 acute events, no patient experienced grade ≥ 3 late effects.

As summarized, despite the relatively few data of particle therapy for glioma, there are also several reflections. Both LGGs and HGGs are extremely heterogeneous populations with differing prognoses. As such, although clinical outcomes were emphasized herein, there is much more to the complete story than survival, which can be influenced by molecular signatures of the tumor, salvage therapies, and other factors. Toxicity reductions are arguably just as important, but still suffer from the dependency on patient selection, regardless of whether the study is retrospective or prospective [39]. Next, although many studies described in this section pertain to dose-escalation, without clear clinical benefit other than inherently faulty comparisons with seminal prospective trials [42, 43], this should still be considered experimental with particle therapy until randomized data prove a benefit. Only then toxicity reductions from particle therapy may be of true clinical benefit. Lastly, despite just one study, the role of particle therapy in re-irradiation cannot be understated, as potentially serious complications may occur to a greater degree using escalated doses (even with particle therapy). However, there are other confounding factors that prevent the generalizability of this statement, such as target margins in the re-treatment setting, availability of high-quality image-guidance, and potential administration of concurrent therapies (e.g. bevacizumab). The ongoing CINDERELLA trial at the University Hospital Heidelberg and Heidelberg Ion-Beam Therapy Center (HIT) is the first study to prospectively evaluate carbon ion re-irradiation (escalating doses up to 48.0 Gy(RBE) in 16 fractions) for recurrent gliomas, and will compare this to fractionated stereotactic photon RT (36 Gy in 18 fractions) [44].

## Concluding remarks

The striking rise of particle therapy across the world necessitates evidence to justify its ever-increasing utilization. Herein, we summarize the current status of these technologies on treatment of both meningiomas and gliomas. Overall, with the notable caveat that the overall quality and quantity of data are low, particle therapy offers significant safety and efficacy with which to treat both neoplasms in either a standard, or less commonly, a dose-escalated setting. Further work must verify and build upon the lessons learned from these data and critically assess whether particle therapy is indeed a necessity in various clinical settings. These data also have implications on the cost-effectiveness of particle therapy [45, 46]. Although a complete discussion is beyond the scope of this article, there may be substantial cost savings associated with a decrease in doses to several OARs in the many survivors of neoplasms discussed herein (e.g. meningioma and LGG). However, a link between dosimetry and clinical toxicity reduction remains to be proven. For instance, preservation of memory and quality of life from decreased hippocampal doses during whole brain RT (a focus of the Radiation Therapy Oncology Group 0933 trial) [47] are both associated with economic cost reductions. Similarly, it may be extrapolated that particle irradiation for various clinical settings, tumor locations, and baseline functionality may have differential likelihoods of having cost-effective RT delivery. However, further data are needed in order to corroborate this notion.

## Abbreviations

3DCRT: 3D conformal RT
CIRT: carbon ion RT
Gy: Gray
HGG: High-grade glioma
IMPT: Intensity-modulated proton therapy
IMRT: Intensity-modulated RT
LGG: Low-grade glioma
OARs: Organs-at-risk
OS: Overall survival
PBT: Proton beam therapy
QOL: Quality of life
RBE: Relative biological effectiveness
RT: Radiation therapy
WHO: World Health Organization
